# The Principles of Hygiene

**Published:** 1902-02

**Authors:** 


THE PRINCIPLES OF HYGIENE. A Practical Manual for Students, Physicians and Health Officers. By D. H. BERGEY, A. M., M. D., First Assistant, Laboratory of Hygiene, University of Pennsylvania. Octavo volume of 495 pages, illustrated. Philadelphia and London: W. B. Saunders & Co. 1901. [Cloth, $3.00 net.]

Preventive medicine assumes an increasingly important place as our knowledge of the causes of disease grows. Now that it has been learned that filth and other distinctly avoidable factors are many times responsible agents rather than Providence, nation, state, and municipality are bestowing more and more attention on all the ways and means of disease prevention. So a call comes for better trained sanitarians and to meet this it comes to pass that medical schools as a whole are taking pains to educate more thoroughly in hygiene. This book is an outgrowth of the practical teaching of hygiene in one of our great universities. As such it may be expected to meet the needs of the student especially. This it does, but at the same time it will be found of value to the practitioner of medicine and the practical sanitarian; and students of architecture who need to consider problems of heating, lighting, ventilation, water supply and sewage disposal, may consult it with profit, giving as it does the sanitarians point of view.

The following chapter headings give some idea of the scope of the work: ventilation, heating, water supply, removal and disposal of sewage, garbage disposal, industrial hygiene, naval hygiene, school hygiene, and the like. Some of these various subdivisions are treated suggestively rather than exhaustively. One wishes at times that the author had gone a little farther. The matter presented, however, in every case represents the latest knowledge upon the topic under discussion.

The metric system of weights and measures is employed throughout the work. The mechanical part of the book is well executed.—M.J.F.

